# Computed Tomography-Measured Cranial Sternal Lymphadenomegaly Is Associated with Elevated C-Reactive Protein in Small Dogs with Non-Neoplastic Disorders

**DOI:** 10.3390/vetsci12040356

**Published:** 2025-04-11

**Authors:** Yutaro Ide, Yu Furusawa, Takeshi Sogawa, Kaori Takahashi, Tomohide Kuramoto, Masashi Takahashi, Naoki Miura

**Affiliations:** 1 Joint Graduate School of Veterinary Medicine, Kagoshima University, Kagoshima 890-0065, Japan; 2 Veterinary Teaching Hospital, Joint Faculty of Veterinary Medicine, Kagoshima University, Kagoshima 890-0065, Japan

**Keywords:** computed tomography, C-reactive protein, lymphadenomegaly, sternal lymphadenomegaly, canine, sternal lymph node

## Abstract

Lymphadenomegaly is well described in dogs with neoplastic diseases but can also reflect inflammation. However, its role in assessments of inflammation is little reported in the veterinary literature. Lymph nodes are most easily assessed superficially through palpation, but computed tomography (CT) enables the evaluation of deep lymph nodes. Accordingly, we investigated associations between the maximal lymph node diameter and plasma concentrations of C-reactive protein (CRP) and white blood cell (WBC) count, as inflammatory markers, targeting three deep lymph nodes (sternal, cranial mediastinal, and internal iliac). We evaluated data from small dogs with non-neoplastic diseases that underwent synchronous CT and blood biochemistry at our institution. We found that the sternal lymph node diameter was significantly greater in dogs with clinically elevated CRP than those with clinically unremarkable CRP (*p* = 0.04) and significantly correlated with CRP (r = 0.32; *p* < 0.01). We found no significant association with CRP for the cranial mediastinal or internal iliac lymph node. Thus, we consider CT-measured sternal lymphadenomegaly a clinically valuable finding for the comprehensive evaluation of systemic inflammation. We anticipate further research may highlight the utility of CT lymph node measurements for such evaluations.

## Simple Summary

Veterinarians often find enlarged lymph nodes when they treat cancer or inflammatory conditions. Deeper lymph nodes can be examined with imaging techniques like computed tomography, but veterinarians still do not know how deeper lymph node enlargement is linked to inflammation. As a preliminary step to investigating this question, we targeted three clusters of lymph nodes (cranial sternal, cranial mediastinal, and internal iliac), and checked how lymph node diameter relates to two parameters that increase at the onset of inflammation (C-reactive protein and white blood cell count), in small dogs with diseases other than cancer. We found that the cranial sternal lymph node was significantly larger when both the C-reactive protein level and white blood cell count were high, and that this lymph node’s size correlated with the protein level overall. However, we found no such pattern for the cranial mediastinal or internal iliac lymph nodes. We thus suggest that measuring the cranial sternal lymph node diameter with computed tomography may be useful for evaluating systemic inflammation. Further research could confirm the broader use of such measurements for assessing inflammation through lymph node size in dogs.

## 1. Introduction

Lymphadenomegaly can occur in dogs suffering from neoplasms [1,2,3], infectious and idiopathic conditions [4,5], and inflammatory disorders [6,7]. In the veterinary literature, enlarged lymph nodes primarily receive attention in oncological studies, particularly those focusing on lymphoma or lymph node metastasis [1,2,3,8,9,10,11]. Non-neoplastic inflammatory disorders have been discussed as differential diagnoses when lymphadenopathy is observed in a review in the human medical literature [12]; however, this issue appears to be little evaluated in the veterinary literature. The paucity of relevant data may reflect challenges in differentiating between inflammatory and neoplastic disorders when lymph node enlargement is observed, considering that inflammation and neoplasia often co-exist, and there is a lack of studies on lymph node enlargement in cancer-free dogs. Veterinary clinicians would clearly benefit from more evidence on the role of lymphadenomegaly in the assessment of inflammatory disorders.

Clinical evaluations of lymph node enlargement typically involve a semi-qualitative judgment based on the palpation of superficial lymph nodes. This approach does not provide any insight into deeper lymph nodes, which may be more sensitive to systemic inflammation due to their anatomical locations. Deep lymph nodes can be evaluated ultrasonographically [13,14], and such techniques have been applied in canine oncological studies. However, factors like the gas-related distortion of sound waves can hinder accurate ultrasonographic visualization of these deeper structures.

Computed tomography (CT) offers a practical, non-invasive imaging modality for evaluating deeper lymph nodes. Unlike ultrasonography, CT provides robust visualization that is unaffected by respiratory gases. It also delivers high-resolution images that quickly and accurately identify lymph node locations [15]. CT has already demonstrated utility for deep lymph node assessments in dogs, by aiding the identification of metastasic cancer in sternal lymph nodes [16], and predictions of metastasis based on tracheobronchial lymph node diameter measurements [17]. It is thus plausible that CT will also have utility for lymph node evaluations in cases of non-neoplastic, systemic inflammation, and generating quantitative data on lymph node geometry that can enhance such evaluations.

One way to contextualize lymphadenomegaly in inflammatory disorders would be to investigate relationships with known markers of systemic inflammation. In both veterinary and human medicine, C-reactive protein (CRP) is widely used as one such marker, and this acute-phase protein also has utility in assessments of tumors and for post-surgical monitoring [18,19,20]. CRP is an acute-phase protein primarily produced by hepatocytes, and it exhibits a dramatic increase during acute inflammation and infection. It is also closely associated with inflammatory cytokines such as interleukin-6 (IL-6) and tumor necrosis factor-alpha (TNF-α). Serum CRP is observably elevated in conditions ranging from acute pancreatitis to chronic inflammatory enteropathy, making it a widely used biomarker for the prognosis and prediction of recurrence [21,22,23]. The relationship between two inflammation-associated parameters—lymph node morphological measurements and CRP levels—thus represents an interesting line of inquiry. However, CRP is a very sensitive, but not selective, marker of inflammation, indicating that it may be useful to include other markers in such investigations. White blood cell (WBC) count is widely used to evaluate inflammation [24]; although, it is much less sensitive than CRP [19]. Nevertheless, incorporating data on both markers has the potential to enhance the interpretation of the results. To the best of the authors’ knowledge, associations between deep lymph node enlargement and markers of systemic inflammation have not previously been investigated in dogs. We hypothesized that deep lymph node measurements would correspond to serum concentrations of inflammatory markers in dogs with inflammatory disorders.

Against this background, in this study, we aimed to investigate associations between lymphadenomegaly and levels of the inflammatory markers, primarily CRP, using CT images of deep lymph nodes (sternal, cranial mediastinal, and iliac lymph nodes) in small dogs with non-neoplastic disorders.

## 2. Materials and Methods

### 2.1. Study Population

For evaluation in this retrospective study, we targeted 665 dogs that underwent CT examinations and hematological and plasma CRP measurements on the same day while receiving medical care at Kagoshima University Veterinary Teaching Hospital (Kagoshima, Japan) between January 2022 and October 2024. The inclusion criteria were a body weight not exceeding 10 kg to minimize any bias in body weight or size (set in reference to a previous report [25]); an age of at least one year; and the presence of tomographically measurable sternal, cranial mediastinal, and/or internal iliac lymph nodes. Dogs were excluded from the study when their cases corresponded to any of the following conditions: diagnosis of a neoplasm, previous treatment for a neoplasm, and any evidence in their medical history that could give rise to suspicion of cancer and/or metastasis. These exclusion criteria were set to ensure that no cases of lymphadenomegaly due to lymph node metastasis or inflammatory reactive hyperplasia associated with cancer were included in this study. Ethics approval was waived for this retrospective study because it did not involve any procedures with experimental animals. It was conducted in accordance with the research ethics by-laws of Kagoshima University. The owner of each dog evaluated in this study had given consent to the use of the relevant data in research at the time of medical examinations.

### 2.2. Evaluation of Lymph Node Images

The dogs had undergone CT imaging with a dedicated 16-helical sliced CT scanner (Aquilion TSX−201A, Toshiba Medical Systems Corporation, Tochigi, Japan), with or without anesthesia. These CT images were acquired with reading and measurement under two conditions (soft tissue condition WL50:WW350; lung condition WL:−50WW:1500). Slice thickness was 1 mm. Blood was collected from each dog on the same day as the CT imaging, for standard blood examinations, which included determining the WBC count with a hematology analyzer (Procyte DX, IDEXX LABORATOIES, Westbrook, ME, USA) and measurement of the CRP concentration in plasma with a clinical chemistry analyzer (Fuji DRI-CHEM NX500, Fujifilm Corporation, Tokyo, Japan).

In this study, the CT images for each dog were retrieved to determine lymph node diameters in three target regions. All images were acquired in DICOM format and were read and measured using the DICOM viewer OsiriX Ver. 5.9 (Pixmeo, Bernex, Switzerland). We targeted the following lymph node clusters: the sternal and cranial mediastinal lymph nodes, which were selected as corresponding to the most commonly identified nodes by CT within the thoracic cavity [15]; and the internal iliac lymph nodes, which were selected based on their presence along abdominal blood vessels (internal iliac artery) and as the most easily identifiable nodes in the abdominal cavity.

Transverse CT images of the thoracic and abdominal cavities of each dog were examined for the target lymph nodes. The sternal, cranial mediastinal, and internal iliac lymph nodes were identified on the relevant CT image as structures of the expected appearance (spherical or oval in shape, with distinct margins) at their expected anatomical locations.

**Figure 1:**
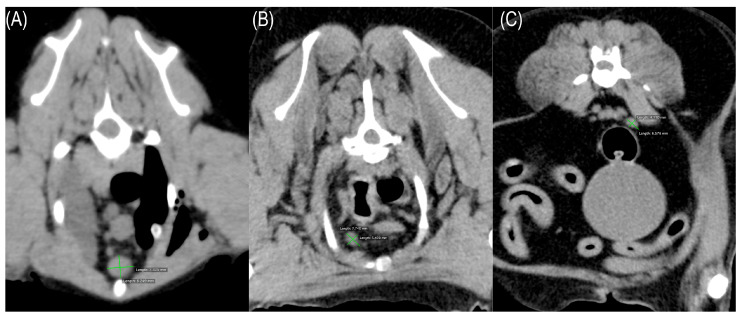
Measurable lymph nodes in computed tomography. Representative examples of clinically enlarged lymph nodes are shown for sternal (**A**), cranial mediastinal (**B**), and internal iliac (**C**) lymph nodes.

When target lymph nodes were identified, two notional, perpendicular lines were plotted through the center of the relevant lymph line extending to its border, and the longer of the two lines was regarded as its maximal diameter. Its measured length was read by the DICOM viewer scale function to the nearest mm and adopted as the value for evaluation (Figure 1). In cases of multiple lymph nodes at a given location, the maximal diameter of the largest node was adopted as the measurement result. Lymph nodes were considered unmeasurable and excluded from the analysis when CT images did not show a structure of the expected appearance at the expected anatomical location, an occupying lesion was detected there, or there was evidence of pleural fluid or ascites. Measurements were performed by a single veterinarian (YI) and confirmed by a second veterinarian (NM) engaged at the Diagnostic Imaging Department of Kagoshima University Veterinary Teaching Hospital.

A measurable lymph node was regarded as a spherical or oval marginal structure with a well-defined boundary at an anatomically expected location. Two notional lines were drawn perpendicular to the boundary line, the longer of the two lines was considered the maximal diameter, and the measured length was taken as the value for evaluation.

### 2.3. Other Data Retrieved

The medical records for each dog with measurable lymph nodes were retrieved, and demographic information (age, breed, sex, and body weight), plasma CRP concentration, and WBC count were collated for evaluation in this study. Each medical record was also checked for any findings of superficial lymph node enlargement on palpation.

### 2.4. Associations Between Lymph Node Enlargement and CRP Level

Lymph nodes were regarded as enlarged when their diameter exceeded a threshold value, set in reference to the literature (sternal lymph node: 7 mm; cranial mediastinal and internal iliac lymph nodes: 6 mm) [15,25,26,27,28,29,30]. Plasma CRP concentrations >0.7 mg/dL were regarded as clinically elevated based on the reference range at our institution. Similarly, a WBC count >16.76 k/dL was also considered to represent a clinically significant elevation.

### 2.5. Statistics Analysis

We evaluated the correlation between lymph node diameter and plasma CRP concentration based on Spearman’s rank correlation coefficient (r-value). We also compared lymph node diameters between dogs with clinically elevated plasma CPR and clinically unremarkable plasma CRP, and between those with clinical elevations in both plasma CRP and WBC count and those with clinically unremarkable values in these parameters. Differences were tested for significance using the Kruskal–Wallis test, and data on lymph node diameters were also analyzed using the Dunn test with the Bonferroni multiple correction as a multiple test. All analyses were performed using the statistical analysis software package GraphPad Prism version 10.0.2 for Windows. (GraphPad Software, La Jolla, CA, USA, www.graphpad.com) A *p* value < 0.05 was considered significant.

## 3. Results

The selection of the study population and the flow of the experiment up to the key findings are illustrated in Figure S1.

### 3.1. Demographic and Clinical Characteristics of the Study Population

A total of 74 dogs were included in the study population (mean age: 10.0 years; mean body weight: 4.6 kg), of which 26 were neutered males, 12 were intact males, 25 were neutered females, and 11 were intact females. The dog breeds encompassed in this study (in descending order of magnitude) were Toy Poodle (*n* = 21), Miniature Dachshund (*n* = 16), Chihuahua (*n* = 14), Shih Tzu (*n* = 4), Papillon (*n* = 3), Miniature Pinscher (*n* = 3), Pekingese (*n* = 2), Yorkshire Terrier (*n* = 2), French Bulldog (*n* = 1), Miniature Schnauzer *(n* = 1), Shiba Inu (*n* = 1), Maltese (*n* = 1), and mixed breed (*n* = 5). The diagnosed conditions for this study population (as determined by the relevant original attending veterinarian) encompassed single or multiple cervical disc herniation (*n* = 3); cystoliths, herniated disc, gallbladder mucocele, small intestinal foreign body, and tracheal collapse (*n* = 2 each); and suspected pancreatitis, lesional adherence to the radial-ulnar ligament, immune-mediated neutropenia, chronic bronchitis, spinal osteoarthritis, rhinitis, encephalitis, idiopathic polyarthritis, cholelithiasis, aortic thrombus, polyarthritis, choledocholithiasis, duodenal polyposis, syncope, uterine cysticercosis, eosinophilic enteritis, laryngeal paralysis, megaesophagus, acute pancreatitis, hepatitis, diaphragmatic hernia, inflammatory polyps, lymphocytic plasmatic rhinitis, and epilepsy (*n* = 1 each). Demographic and diagnostic data are presented in Table 1.

For these 74 dogs, the sternal, cranial mediastinal, and internal iliac lymph nodes were measurable in 68, 69, and 73 cases, respectively. Imaged lymph nodes were mostly of predefined morphology and showed the expected attenuation on CT (with Hounsfield unit [HU] values within the expected range). In some cases, low density areas were visible within the lymph nodes, but they did not affect determination of the nodal morphology or diameter. Representative CT images of measurable lymph nodes are shown in Figure 1.

Based on the measured diameter, the subpopulations with enlarged lymph nodes were as below.

A total of 4/68 dogs (5.9%) showed enlarged sternal lymph nodes (7.4 to 8.1 mm). These dogs were two neutered females, one intact female, and one intact male. Individually, these dogs were a Miniature Dachshund (age: 14 year 5 months, body weight: 9.3 kg) diagnosed with a small intestinal foreign body, a Toy Poodle (age: 10 years, body weight: 2.94 kg) diagnosed with diaphragmatic hernia, a Toy Poodle (age: 10.8 years, body weight: 2.0 kg) diagnosed with lung lobe torsion, and a mixed breed dog (age: 5.5 years, body weight: 2.5 kg) diagnosed with gallbladder mucocele.

A total of 9/69 dogs (13.0%) showed enlarged cranial mediastinal lymph nodes (range: 6.2 mm to 9.3 mm). These dogs comprised one intact and three neutered males (total: *n* = 4; age: 6.1 years to 12.8 years) and one intact and four neutered females (total: *n* = 5; age: 4.3 years to 16.5 years). Individually, these dogs were three Toy Poodles, three Chihuahuas, one Shih Tzu, and one Miniature Dachshund. The diagnosed conditions for these dogs were gallbladder mucocele (*n* = 1), disc herniation (*n* = 2), polyarthritis (*n* = 2), tracheal collapse (*n* = 1), reactive follicular hyperplasia (*n* = 1), lung lobe torsion (*n* = 1), and extrahepatic bile duct obstruction (*n* = 1).

A total of 15/73 dogs (20.5%) showed enlarged internal iliac lymph nodes (range: 6.0 to 8.1 mm). These dogs comprised three intact and three neutered males and two intact and seven neutered females (age: 4.5 years to 16.2 years). The diagnosed conditions for these dogs were disc herniation (*n* = 3), polyarthritis (*n* = 2), immune-mediated neutropenia (*n* = 1), gallbladder mucocele (*n* = 1), inflammatory polyp (*n* = 1), arterial thrombosis (*n* = 1), bladder stones (*n* = 1), spinal cord compression (*n* = 1), cerebral infarction (*n* = 1), hepatic encephalopathy (*n* = 1), and acute pancreatitis (*n* = 1).

Blood examinations revealed that 29/74 dogs (39.2%) had clinically elevated plasma CRP. Furthermore, 14/74 dogs (18.9%) showed an elevated WBC count (Table 1). Of these, 11/74 dogs (14.9%) showed both clinically elevated plasma CRP and WBC counts. The diagnosed conditions for these dogs were gallbladder mucocele (*n* = 3), small intestinal foreign body (n = 2), hepatic encephalopathy (*n* = 1), acute pancreatitis (*n* = 1), pyometra (*n* = 1), pneumonia (*n* = 1), arterial thrombosis (*n* = 1), and eosinophilic enteritis (*n* = 1).

Palpably enlarged superficial lymph nodes were only noted in one case (an instance of enlarged mandibular lymph nodes).

### 3.2. Deep Lymph Node Evaluation in Dogs with Elevated Plasma CRP

**Figure 2:**
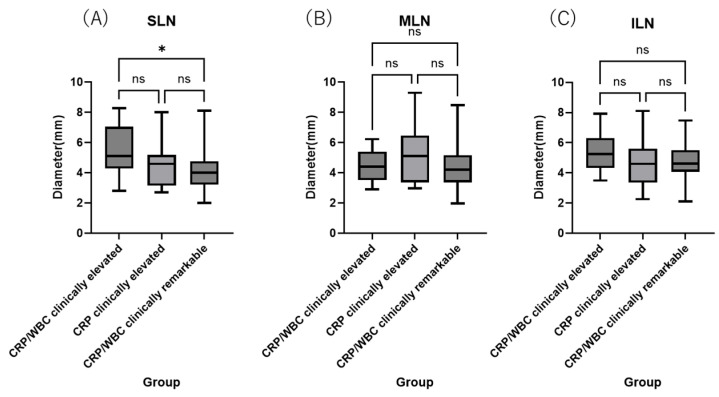
Lymph node diameters in dogs with clinically elevated CRP and WBC, clinically elevated CRP and normal WBC, and clinically unremarkable CRP and WBC. (**A**) sternal lymph nodes; (**B**) cranial mediastinal lymph nodes; (**C**) internal iliac lymph nodes. *: significant different, ns: not significant.

To evaluate potential associations with systemic inflammation, we compared lymph node diameters between dogs with clinically elevated CRP concentrations and WBC counts, those with only clinically elevated CRP (but normal WBC counts), and those with clinically unremarkable plasma CRP concentrations and WBC counts. Dogs with a clinically elevated plasma CRP plus WBC count showed significantly greater sternal lymph node diameters than dogs with clinically unremarkable values in these parameters (*p* = 0.04). We found no significant difference in the sternal lymph node diameter for dogs with only elevated plasma CRP (although they tended to show larger diameters for this lymph node), or for any comparison with the cranial mediastinal (*p* = 0.228) or internal iliac (*p* = 0.225) lymph nodes (Figure 2).

Lymph node diameters (mm) in dogs with clinically elevated CRP and WBC, clinically elevated only CRP, and clinically unremarkable CRP and WBC were tested. Based on the reference values testing companies provided, CRP was categorized as clinically unremarkable at concentrations <0.7 mg/dL, or elevated at concentrations of 0.7–7.0 < mg/dL. Similarly, the WBC count was categorized as clinically unremarkable at <16.76 k, or elevated at 16.76 k or above.

### 3.3. Associations Between Lymph Node Diameter and Plasma CRP in Correlation Coefficient

**Figure 3:**
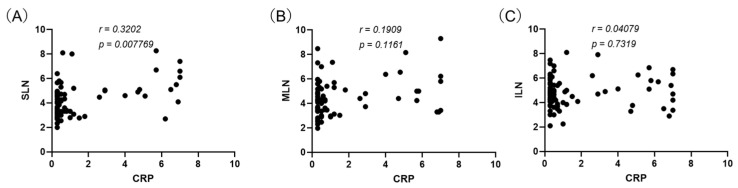
Lymph node diameter relation with CRP concentration (Spearman’s rank correlation coefficient). (**A**) sternal lymph nodes; (**B**) cranial mediastinal lymph nodes; (**C**) internal iliac lymph nodes.

Correlation analyses targeting the lymph node diameter and CRP revealed a weak positive correlation for the sternal lymph nodes (r = 0.320, *p* < 0.001).(Figure 3A) On the other hand, no correlation with CRP was found for the cranial mediastinal lymph nodes (r = 0.190, *p* = 0.116) or internal iliac lymph nodes (r = 0.041, *p* = 0.732).(Figure 3B,C).

To evaluate the effect of body weight on the evaluation in this study, correlations with CRP level were determined again for lymph node diameters normalized by correction with body weight indices. Equation (1) below was used for body weight adjustment: LN_dia_ represents the maximum diameter of each lymph node, and BW*^x^* denotes the body weight raised to a given power.

(1) $$\frac{LN_{dia}}{BW^{x}}$$

The strongest correlation was observed for the cranial sternal lymph node when using a correction index of BW^0.1^ (r = 0.4195, *p* < 0.01). The cranial mediastinal lymph node showed a significant positive correlation (r = 0.27, *p* = 0.0244). However, the internal iliac lymph nodes did not exhibit any significant correlation under any of the applied corrections (*p* = 0.4646) (Figure S2).

## 4. Discussion

In this study, we investigated associations between CT-measured canine lymphadenomegaly and elevated CRP in dogs without neoplastic disorders, targeting deep lymph nodes (cranial mediastinal, sternal, and internal iliac lymph nodes), based on our hypothesis that increased deep lymph node diameters would reflect increased levels of the acute-phase protein in conditions of systemic inflammation. To strengthen the interpretation of our data, our comparisons targeted dogs with elevations in both the plasma CRP and WBC count, as well as those with elevations in CRP only. To the authors’ knowledge, this is the first report on such lymph node-related associations in the canine medical literature.

In our key findings, somewhat surprisingly, only one of our target lymph nodes, the sternal lymph nodes, showed associations with CRP. Sternal lymph node diameter was significantly correlated with plasma CRP concentration overall, and was significantly greater in dogs with clinically elevated plasma CRP and WBC counts than in dogs with clinically unremarkable values in these two parameters. A tendency to find the same difference was noted in comparisons limited to elevations in plasma CRP only, but this difference was not significant. Our findings are consistent with reports that primary infectious or inflammatory diseases represent causative conditions in dogs and cats presenting with radiologically enlarged lymph nodes, although neoplastic diseases represent the most prevalent diagnosis [26,31]. Furthermore, sternal lymphadenomegaly has been reported in a dog showing an inflammatory response to systemic Geomyces infection [32], a dog with Pneumocystis carinii infection [33], and a dog with steroid-responsive meningitis-arteritis presenting further complications [34]. Taken together, these results indicate that a link between sternal lymph enlargement and systemic inflammation is plausible.

The dogs with enlarged sternal lymph nodes in this study had been diagnosed with various conditions. Of these diagnosed conditions, gallbladder mucocele (*n* = 1 in this study) has previously been linked with high CRP levels [35,36], and intestinal obstruction due to foreign bodies (*n* = 1) can cause damage to the intestinal wall, resulting in systemic acute inflammation [37]. The other two conditions (diaphragmatic hernia and lung lobe torsion, each *n* = 1) are not typically regarded as direct causes of systemic inflammatory reactions. However, they are reportedly associated with elevated WBC counts [38,39,40], so indirect, systemic inflammatory responses may be feasible in these cases. In short, we cannot rule out sternal lymph node enlargement reflecting a local reaction in lung tissue in the lobular torsion case. Still, we consider that sternal lymph node enlargement where lymph nodes adjacent to an abdominal lesion were not swollen may well reflect a systemic, and not a local, reaction.

In contrast to our results for the sternal lymph nodes, this study provided no evidence that cranial mediastinal or internal iliac lymph node enlargement reflects systemic inflammation, as we found no significant correlation with CRP, or significant differences between dogs with clinically elevated and unremarkable CRP.

Limiting this study population to dogs weighing less than 10 kg reduced, but did not fully eliminate, the potential for variation in body weight to influence the evaluation of the study results. However, based on the results of our supplementary experiments with normalized lymph node diameters, we consider any such influence to be minimal. When using a lower correction index for body weight, the statistical significance of the correlation for the cranial sternal lymph became stronger, and at a higher correction index—thereby giving a greater weighting to body weight—the statistical significance of this correlation was weakened. Additionally, the cranial mediastinal lymph node exhibited a significant positive correlation when body weight correction indices of approximately 0.1–0.3 were applied. However, in the case of the cranial mediastinal lymph nodes, there was no change in significance for the correlation, and it remains unclear whether they are more susceptible to the effects of body weight. Nevertheless, it is important to note that even without correction, the diameter of the cranial sternal lymph node reliably reflects the CRP level.

The cranial mediastinal lymph nodes are located within the thoracic cavity, similarly to the sternal lymph nodes; however, they drain the neck and chest regions, whereas the sternal lymph nodes drain the local skin, mammary glands, thoracic and abdominal walls, and the diaphragm [41], which may explain the difference in results we found here. The internal iliac lymph nodes are located within the hypogastric lymphosome, which occupies a relatively small region at the caudal end of the abdominopelvic cavity, indicating that these lymph nodes are potentially less sensitive to systemic inflammatory responses than the nodes in centers we investigated in the thoracic cavity. Some caution is required in interpreting these results, though, because of the possibility that localized inflammation-induced lymph node enlargement may have occurred without CRP elevation in some cases. However, it is biologically feasible that the sternal lymph nodes, which receive lymphatic inflow from locations across the abdominal and thoracic cavities, are sensitive to systemic inflammation, in ways that other lymph nodes targeted in this study (and which have more localized lymphatic inflows) are not. Furthermore, it is noteworthy that almost no superficial lymph enlargement was reported in the dogs in this study, indicating potentially greater sensitivity for these deep lymph nodes, and that images of deep lymph node enlargement may provide more useful clinical information on the lymphatic system than findings solely based on palpation.

Overall, in this study, the proportion of dogs with clinically enlarged lymph nodes was greatest for the internal iliac lymph nodes, for which no correlation with CRP was noted. Since the internal iliac lymph nodes primarily drain the caudal portion of the abdominal wall, and intra-abdominal conditions were prevalent in our study population, we consider that enlargement of these lymph nodes may have mainly reflected localized inflammation, potentially masking any systemic inflammatory effects for this lymph node through influence on the observed correlations.

Viewing our findings holistically, we recommend clinicians observing enlarged sternal lymph nodes to consider further diagnostic tests to rule out or confirm inflammatory, as well as neoplastic, disorders. In this study, we used CT as the imaging modality because of its excellent spatial resolution and the fact that it allows the evaluation of multiple lymph nodes in serial images. However, ultrasonography also reportedly enables evaluations of sternal lymph nodes [13,14], so it may be more suitable for screening. CT is then used as an auxiliary tool for confirmation and more detailed evaluation.

This study has several limitations. Being a retrospective study, we could not directly confirm the pathological status of the lymph nodes; for superficial lymph nodes, we relied solely on information recorded in the medical records indicating whether they were enlarged. Additionally, our data were collected from dogs at a single time point—specifically, upon referral to our hospital—preventing us from exploring any temporal aspects of the relationship between CRP levels and lymph node diameter. Understanding this relationship over time would be valuable, particularly since CRP functions as an acute-phase protein. Furthermore, as stated above, CRP is not a specific marker of systemic inflammation, which limits the conclusions we can draw about sternal lymph node enlargement as an indication of such inflammation. However, including dogs in which an elevated WBC count accompanied elevated CRP in a separate group for comparison adds a greater degree of reliability to the judgments of inflammatory state in this study population. It should also be noted that the dogs in this study were free of tumors, and had not recently undergone surgery. Although this acute-phase protein and WBC count may not totally specifically reflect an inflammatory condition, we consider that its utility for quantitative assessments makes it a biologically relevant parameter. Nevertheless, a more robust determination of systemic inflammation covering pathological evaluations (such as lymph node biopsy results) and more comprehensive assessments involving multiple inflammatory cytokines would have strengthened our results, and should be considered in future research. Inferring systemic inflammation from only CRP and WBC may involve some risk of misinterpretation of the lymph node findings, when an evaluation does not include adjacent lymph nodes and the anatomical and functional relationship to the relevant lymphosome. However, we consider that the correlation between sternal lymph node enlargement and our markers of inflammation (CRP and WBC) is at the very least an association of interest, given the likely presence of inflammatory conditions in the abdominal, rather than the thoracic, region.

## 5. Conclusions

We demonstrated that the CT-measured maximal sternal lymph node diameter has potential utility in evaluating systemic inflammation, based on its association with the measured plasma CRP concentration (also augmented with results for the WBC count), in small dogs free of neoplastic disease. Considering that sternal lymph nodes were enlarged in the absence of any thoracic disease (in the region drained by these lymph nodes), we postulated the presence of a condition that may cause systemic inflammation. We suggest that the imaging evaluation of sternal lymph nodes with CT or other technologies may have utility for identifying dogs with suspected inflammatory conditions, and informing clinical decisions on further diagnostic testing.

## Figures and Tables

**Table 1:** Demographic and clinical characteristics of small dogs free of neoplasms.

Parameter	Value
Study population	*n* = 74
Male/Female	*n*= 38/*n* = 36
Intact/Neutered	*n* = 23/*n* = 51
Age	Mean 10.0 y (max–min: 18.5 y–1.3 y 95%CI: 11 y–8.9 y)
Sternal lymph nodes	
No. measurable	*n* = 68
Mean diameter	4.4 mm (max–min: 8.3–2 95%CI: 4.7–4.0)
Clinically enlarged	4 (>7 mm)
Cranial mediastinal lymph nodes	
No. measurable	*n* = 69
Mean diameter	4.5 mm (max–min: 9.3–2 95%CI: 4.9–4.1)
Clinically enlarged	9 (>6 mm)
Internal iliac lymph nodes	
No. measurable	*n* = 73
Mean diameter	4.8 mm (max–min: 8.1–2.1 95%CI:5.1–4.5)
Clinically enlarged	15 (>6 mm)
CRP	1.9 mg/dL (max–min: 7.0–0.3 95%CI: 2.4–1.31)
Clinically elevated	29 (>0.7 mg/dL)
WBC	13.9 mg/dL (max–min: 50.9–4.9 95%CI: 16.1–11.7)
Clinically elevated	14 (>16.76 k/μL)

## Data Availability

The raw data supporting the conclusions of this article will be made available by the authors upon request.

## Supplementary Materials

The following supporting information can be downloaded at: https://www.mdpi.com/article/10.3390/vetsci12040356/s1, Figure S1: Participant flow in study of lymph node diameters. Figure S2: Body weight-adjusted lymph node diameter relation with CRP concentration (Spearman’s rank correlation coefficient).
