# Supplementary material for: Computed Tomography-Measured Cranial Sternal Lymphadenomegaly Is Associated with Elevated C-Reactive Protein in Small Dogs with Non-Neoplastic Disorders
Source: Vet Sci. 2025 Apr 11;12(4):356. doi: 10.3390/vetsci12040356 (PMC12030960; doi:10.3390/vetsci12040356)
Supplement: Supplementary file 1 [file vetsci-12-00356-s001.zip › vetsci-3545584-supplementary.pdf]

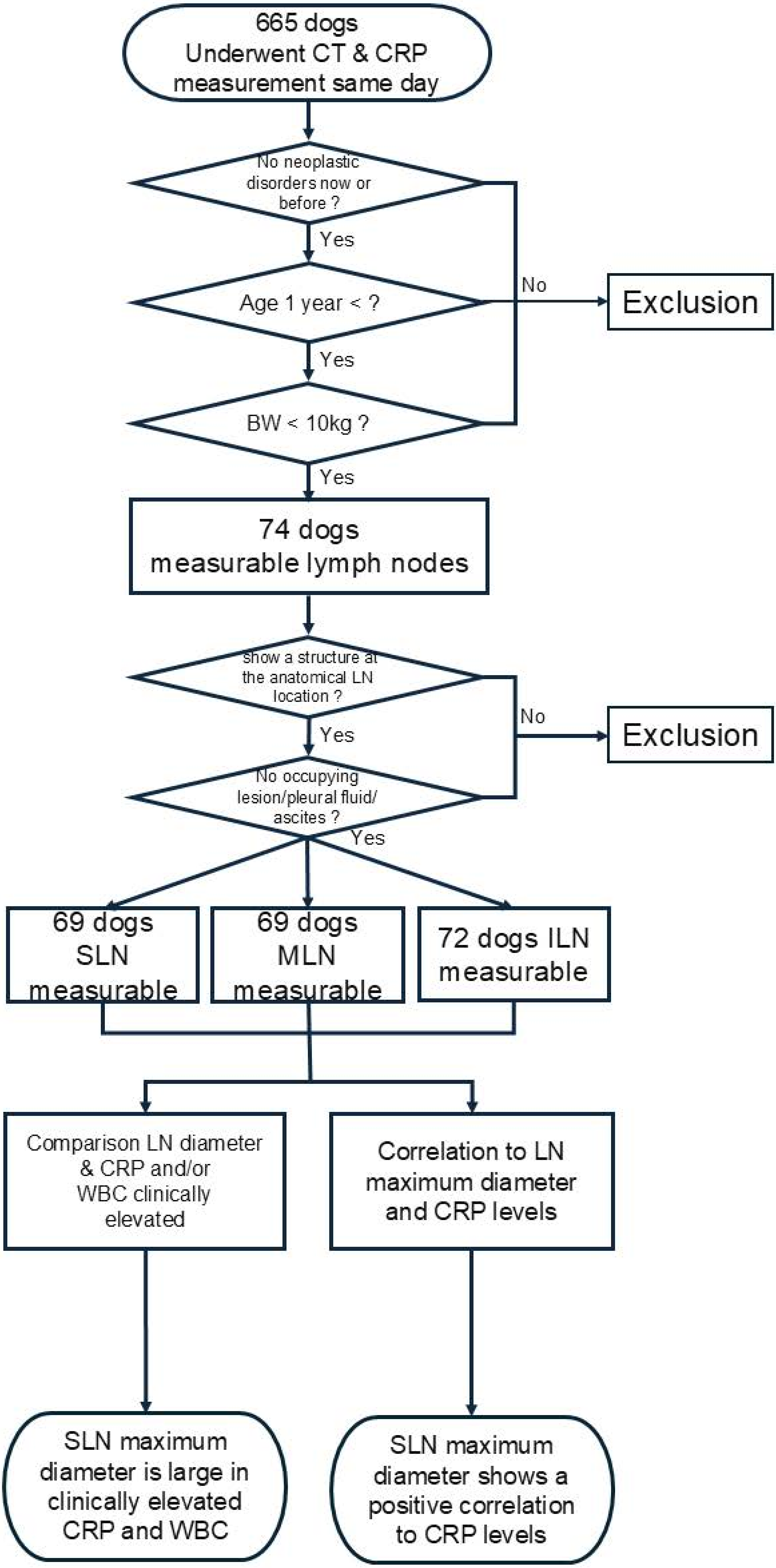

Figure S1 Participant flow in study of lymph node diameters
CT; Computed tomography, CRP; C-reactive protein, BW; Body weight, LN; Lymph node, SLN; Sternal lymph node, MLN; cranial mediastinal lymph node, ILN; internal iliac lymph node, WBC; white blood cell count

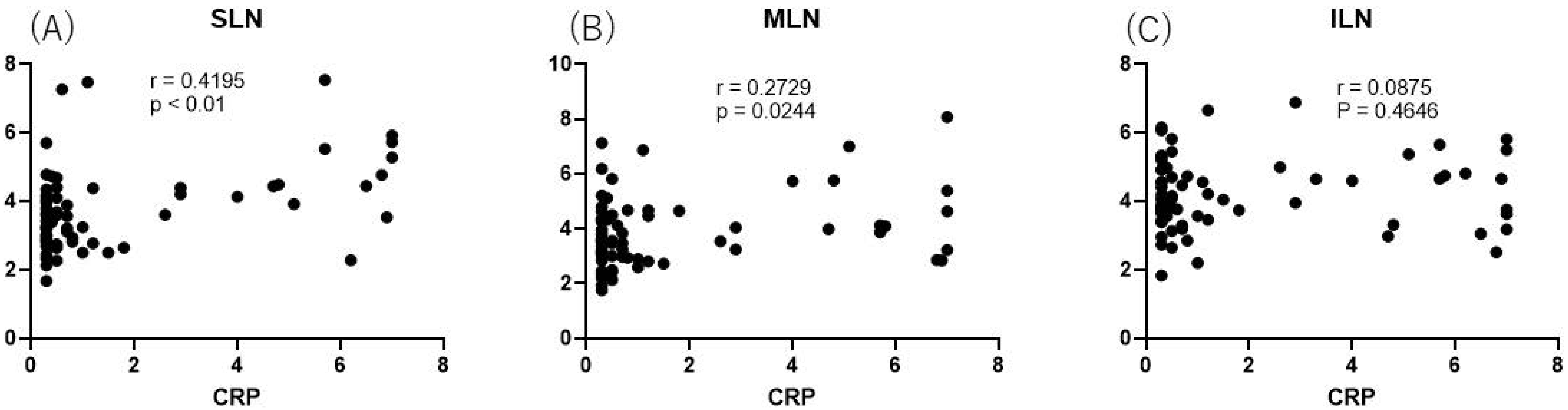

Figure S2 Body weight-adjusted lymph node diameter relation with CRP concentration (Spearman's rank correlation coefficient).
A: sternal lymph nodes; B: cranial mediastinal lymph nodes; C: internal iliac lymph nodes
